# Effect of precipitation on respiration of different reconstructed soils

**DOI:** 10.1038/s41598-020-63420-x

**Published:** 2020-04-30

**Authors:** Na Lei, Jichang Han

**Affiliations:** 1 Shaanxi Provincial Land and Engineering Construction Group CO., LTD., No.7 Guangtai Road, Xi’an, Shaanxi Province, China; 2 Institute of Soil and Water Conservation, State Key Laboratory of Soil Erosion and Dryland Farming on the Loess Plateau, Northwest A&F University, No.3 Taicheng Road, Yangling, Shaanxi Province, China

**Keywords:** Agroecology, Environmental impact

## Abstract

Respiration and hydrothermal characteristics of four reconstructed soils in barren gravel land at a site in Shaanxi Province were monitored before, during, and after two precipitation events. Both precipitation events significantly reduced soil temperature but there were great fluctuations in temperature after the second precipitation event. Moreover, precipitation increased the moisture content of the reconstructed soils. Before the first precipitation event, the soil volumetric water content was relatively stable, while it gradually decreased before the second precipitation event. The first precipitation event significantly stimulated the respiration rate for all reconstructed soils, while the second precipitation event generally inhibited it, especially during the precipitation event. The key factors influencing respiration for different reconstructed soils were different between the precipitation events. When soil volumetric water content showed persistent variation before precipitation, soil moisture was the most influential factor. In contrast, if water content was stable, soil temperature was more influential. Soil moisture and temperature jointly influenced soil respiration before, during, and after the precipitation event, while soil moisture was always the most influential factor after precipitation.

## Introduction

Soil respiration is the main component of the carbon cycle in terrestrial ecosystems. It is the second largest source of carbon emissions after atmospheric photosynthesis between the atmosphere and terrestrial ecosystems^1,2^. Small changes in soil respiration significantly affected global carbon dynamics^3^. A detailed analysis of the dynamics of soil respiration and its controlling factors is of great significance for accurately predicting changes in the carbon cycle under future climate change conditions^4^. Soil temperature and soil moisture are the most important environmental factors affecting soil respiration^5,6^, and there is an interaction between the effects of soil temperature and soil moisture on soil respiration. Under conditions of future climate change, precipitation will change greatly, and the frequency of drought and extreme precipitation will increase^7^. There are two different views on how precipitation might affect soil respiration. One is that precipitation will strongly and quickly stimulate soil respiration. This pulse is called the ‘Birch effect’^8^. However, it has been shown that the ‘Birch effect’ does not apply to all soils, and that for moist to wet soils, precipitation will instead inhibit soil respiration^9^. The effect of precipitation on soil respiration is affected by environmental conditions such as soil temperature and moisture, and there is complex relationship of soil respiration with soil temperature and moisture. Several previous studies have demonstrated that there are many factors that could influence the response of soil respiration to precipitation events, including, both antecedent soil moisture and precipitation conditions^10^, different soil temperature and moisture conditions^11^, temperature sensitivity^12^ and different precipitation^13^.

With the reduction of high-quality reserve cultivated land resources, the current land remediation has evolved from simple land levelling and construction of supporting facilities to soil rebuilding in China. Soil rebuilding of barren beach land was first implemented in a pilot project in Meixian County, Baoji City, Shaanxi Province. The implementation scale of the barren land improvement project has reached 1204.08 ha in this county, with 846.75 ha of newly created cultivated land. At the same time, it has been widely promoted in Huayin City and Taibai County, Baoji City. Reconstructing plough horizons by covering the soil is a key technology for rebuilding in barren gravel land. The engineering innovation lies in the addition of soft rock, shale, and other soil-forming materials together with sand, meteorite, and other improved materials at specific ratios^14–20^, aiming to improve the quality, nutrient content, and texture of cultivated land through remediation^21–23^. The reconstruction of the soil in barren gravel land by addition of different materials would inevitably affect the regional climate. Precipitation as a disturbance factor is important for accurately estimating the soil respiration of the reconstructed soils. In the context of global warming, research on the effects of precipitation on soil respiration of reconstructed soils is still rare. Furthermore, the reconstructed soils will become the main direction for land remediation in the future and an important means to expand the available pool of cultivated land resources. Therefore, studying the effects of precipitation on soil respiration of reconstructed soils under different materials can help to clarify the mechanisms of soil respiration and dynamic change in reconstructed soils under precipitation conditions, which can provide data to support a theoretical basis for accurate assessment of regional CO_2_ emissions and the development of more accurate CO_2_ emission reduction measures. Therefore, it is of great practical significance to study the response mechanisms of reconstructed soils to precipitation. In this study, the effects of precipitation on soil hydrothermal factors and changes in respiration of reconstructed soils were analysed before, during, and after two different precipitation events for four reconstructed soils under natural conditions at a site in Meixian County, Shaanxi Province.

## Materials and Methods

### Overview of test plots

The test plot was located in Shangwang Village, Tangyu Town, Meixian County, Baoji City, Shaanxi Province (107°53′50′′E, 34°8′33′′N), a demonstration area for the barren gravel land remediation project. The total area is 8.00 ha, and the newly added cultivated land is 6.80 ha. Four materials, namely soft rock, sand, shale, and meteorite were selected, crushed through a 10 mm sieve, disinfected, sterilized, and mixed with the soil to form a mixed layer (30 cm) of meteorite, shale, sand and soft rock, and soil. Lou soil, which is the local common soil type, was used as the base soil. Finally, four reconstructed soils were formed: (1) gravel + meteorite + lou (hereafter referred to as ‘meteorite’); (2) gravel + shale + lou (‘shale’); (3) gravel + sand + lou (‘sand’); and (4) gravel + soft rock + lou (‘soft rock’). The amount of meteorite, shale, sand, and soft rock was 1 × 10^−3^ m^3^/m^2^. The dimensions of all test plots were 20 × 30 m^2^. The vegetation in the test plots consisted of tall fescue (*Festuca arundinacea*). The physical and chemical properties of the test plots are shown in Table 1. Three soil respiration rings (inner diameter 10 cm) were buried in each of the four test plots, ensuring that the tops of the rings were 2 cm above the ground. All vegetation (excluding roots) was removed from the soil within the respiration rings to ensure no vegetation growth in the respiration rings during the entire observation period.

**Table 1:** Basic physical and chemical properties of four reconstructed soil mass at 0~20 cm depth.

Detection Indicator		Reconstituted soil mass types			
		Meteorite	Shale	Sand	Soft rock
pH		8.55	8.49	8.51	8.49
Organic carbon (g·kg^−1^)		3.41	3.75	3.7	4.77
Total nitrogen (g·kg^−1^)		0.56	0.36	0.44	0.48
Available phosphorus (mg·kg^−1^)		12.93	26.33	27.27	21.7
Available potassium (mg·kg^−1^)		136.96	130.15	115.54	111.65
Size grading	<0.002 mm	16.47	16.88	15.17	17.85
	0.002~0.05 mm	79.87	76.09	79.99	79.22
	>0.05 mm	6.04	7.03	4.84	2.93

### Soil respiration

Soil respiration changes were measured for five days before, during, and after each precipitation event. The representative precipitation of Meixian County was selected twice, five days before precipitation and five days after precipitation, and soil respiration in all rings was measured. The first precipitation event occurred on April 2, 2018 and was a short-term precipitation event with alternating wet and dry conditions, in which 42.8 mm of rain fell. The second precipitation event occurred on July 2 − 13, 2018, during which 114.7 mm rain fell. The amount of precipitation was measured twice a day at 9:30–11:00 in the morning and 14:30–16:00 in the afternoon.

Soil respiration measurements were performed using a soil carbon flux measurement system (LI-8100, LI-COR Biosciences, Lincoln, NE, USA) equipped with a survey chamber of 20 cm in diameter and equipped with an auxiliary sensor connected to the main unit^24–26^. The sensor can be connected to up to four thermocouples (three input voltages and one soil water content channel). When measuring soil respiration, the respiration chamber was placed on the buried soil respiration ring to ensure that the connection between the respiration ring and the soil respiration chamber was sealed, and the electronic temperature probe connected to the sensor. Moreover, the time domain reflectometry probes were inserted vertically into the soil near each respiration ring to measure soil carbon flux, soil temperature at 5 cm, and water content at 10 cm. For each ring, measurements were taken three times and the measurement time was 4 min.

The precipitation data were mainly recorded automatically by the HOBO weather station (Onset Computer Corporation, Bourne, MA, USA), installed 3 m outside the test plot area^27,28^. The weather station set the measurement time and measurement parameters according to the required meteorological factor data.

### Data analyses

One-way ANOVA was used to analyse differences in soil temperature, soil volumetric water, and soil respiration of the four reconstructed soils. All statistical tests were carried out using SPSS software (version 16.0; SPSS Inc., Chicago, IL, USA). Nonlinear regression was used to assess the relationship between soil respiration and hydrothermal influence factors of the four reconstructed soils, and Q_10_ was estimated. The relationship between soil respiration and soil temperature was fitted by an exponential model (1) and the relationship between soil respiration and water content was fitted by a quadratic curve model (2). Additionally, the relationship between soil respiration and both soil temperature and soil volumetric water content was fitted by a power-index model (3):

1 $${{R}}_{{S}}={a}\,{{\rm{e}}}^{{bT}},\,{{Q}}_{10}={e}^{10b}$$

2 $${R}_{S}=a{{\rm{w}}}^{2}+b{\rm{w}}+c$$

3 $${R}_{S}=a{{\rm{e}}}^{b{\rm{T}}}\,{{\rm{w}}}^{c}$$

where R_S_ is the soil respiration rate (μmol m^−2^ s^−1^); T is the soil temperature (°C); w is the soil volumetric water content (%); *a*, *b*, and *c* are the model parameters, and Q_10_ is the sensitivity coefficient of soil respiration, which refers to the change in entropy of soil respiration rate when the soil temperature rises by 10 °C.

## Results

### Changes in hydrothermal factors before, during, and after precipitation

#### Soil temperature

**Figure 1:**
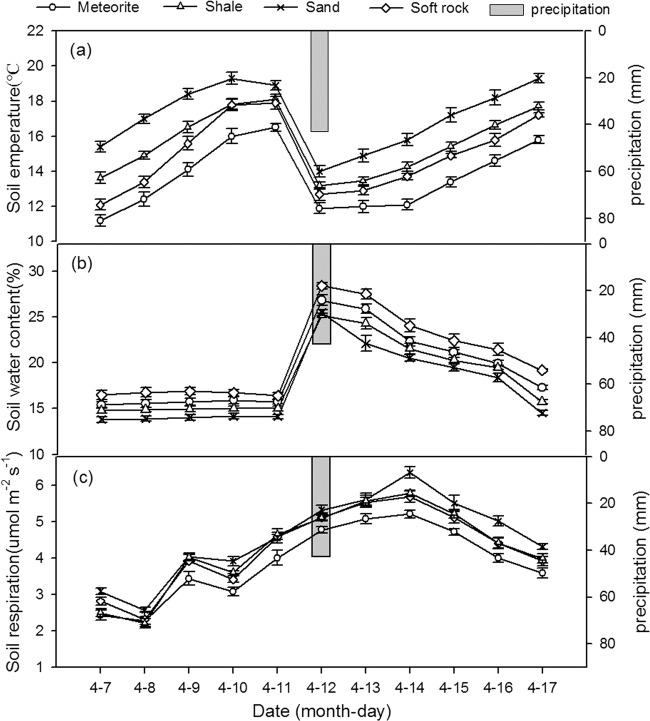
The change trend of temperature (**a**), volumetric water content (**b**) and respiration rate (**c**) of the four kinds of reconstructed soils during the first rainfall.

Figure 1(a) shows the change in soil temperature for 11 days (i.e. five days before, one day during, and five days after the first precipitation event). Precipitation significantly reduced soil temperature. During the observation period, temperature of all reconstructed soils increased before and after precipitation, only decreasing during the precipitation event. Relative to one day before precipitation, temperature of meteorite, shale, sand, and soft rock reconstructed soils decreased by 27.9%, 27.2%, 25.9%, and 29.1%, respectively. The temperature of reconstructed soils gradually increased before precipitation, and the upward trend decreased on the fifth day. The sand reconstructed soil even showed a decreasing trend. After precipitation, the temperature rose every day until pre-rainfall levels were restored. The temperature was not significantly different among the four reconstructed soils before precipitation. During and after precipitation, the reconstructed soils with added meteorite were significantly lower than the other reconstructed soils (*p* < 0.05), the reconstructed soils with added sand were significantly higher than the other soils (*p* < 0.05), but the reconstructed soils with added shale and soft rock did not show a significant difference (*p* > 0.05).

**Figure 2:**
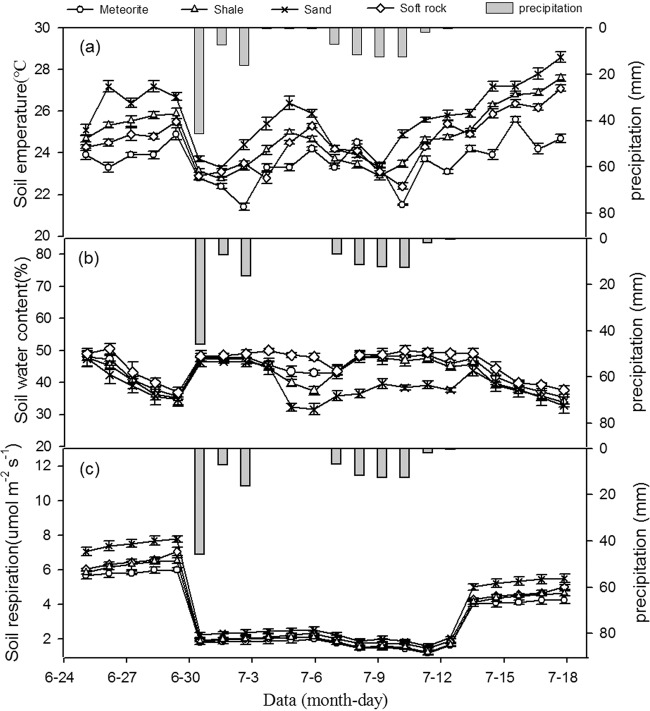
The change trend of temperature (**a**), volumetric water content (**b**) and respiration rate (**c**) of the four kinds of reconstructed soils during the sencond rainfall.

Figure 2(a) shows the change in soil temperature for 22 days (i.e. five days before, 12 days during, and five days after the second precipitation event). The precipitation significantly reduced soil temperature. During the observation period, the temperature of four reconstructed soils increased before and after precipitation, but with great temperature fluctuations after precipitation. Relative to one day before precipitation, average soil temperature decreased 7.3%, 8.0%, 7.4%, and 6.8% during the precipitation in meteorite, shale, sand, and soft rock, respectively. Before the second precipitation event, the reconstructed soils with added sand were significantly higher than the other soils (*p* < 0.05). The temperatures of the reconstructed soil with added shale and soft rock were not significantly different (*p* > 0.05). During the precipitation event, the temperature differences of the four reconstructed soils were not significant (*p* > 0.05). After precipitation, the reconstructed soils with added shale were significantly higher than the soils with added meteorite (*p* < 0.05).

#### Soil water

Figure 1(b) shows the change in soil volumetric water content for 11 days (i.e. before, during, and after the first precipitation event). Precipitation increased water content of the reconstructed soils. During the observation period, the soil volumetric water content was relatively stable before precipitation in all soils. During precipitation, soil volumetric water content significantly increased. The volumetric water content of the meteorite, shale, sand, and soft rock reconstructed soils increased by 41.6%, 27.2%, 26.0%, and 29.1% relative to one day before precipitation, respectively. After precipitation, the soil volumetric water content gradually decreased. The volumetric water content of meteorite, shale, sand, and soft rock reconstructed soils adding were significant different to each other before precipitation (*p* < 0.05) (soil volumetric water content of: soft rock> meteorite> shale> sand). There were no significant differences in soil volumetric water content among the four reconstructed soils during and after precipitation (*p* > 0.05).

Figure 2(b) shows the change in soil volumetric water content for 22 days (i.e. before, during and after the second precipitation event). During the first five days of the second precipitation event, the average volumetric water content of the meteorite, shale, sand, and soft rock soils was 41.9%, 41.0%, 39.8%, and 43.9%, respectively. The average volumetric water content of each soil increased to 46.5%, 45.1%, 39.6%, and 48.5%, respectively, and then gradually decreased. The average volumetric water content of the meteorite, shale, sand, and soft rock reconstructed soils during the five days after precipitation was 40.1%. 38.4%, 37.8%, and 42.0%, and decreased to 35.4%, 33.9%, 32.8%, and 37.6%, respectively, by the fifth day after precipitation. There was no significant difference in soil volumetric water content of the four reconstructed soils before or after precipitation (*p* > 0.05). During the second precipitation event, the soil water content of the sand reconstructed soils was significantly lower than the other reconstructed soils (*p* < 0.05). During the observation period, the volumetric water content of all reconstructed soils decreased gradually five days before precipitation. During the precipitation event, the precipitation first increased, then gradually decreased, then increased again and gradually stabilized; this was significantly related to the total precipitation time and quantity. On July 2, after 45.7 mm precipitation, soil volumetric water content increased significantly. From July 3 to 4, precipitation continued, the volumetric water content was relatively stable, and the frequent precipitation caused the soil water content to accumulate and saturate. From July 5 to 7, there was little precipitation and the soil volumetric water content began to decline, and on July 8, the precipitation increased, causing the soil volumetric water content to start increasing, until gradually stabilizing. The volumetric water content began to decrease after precipitation.

### Changes in soil respiration before, during, and after precipitation

Figure 1(c) shows soil respiration for 11 days, i.e. before, during, and after the first precipitation event. During the first five days, the average respiration rate of meteorite, shale, sand, and soft rock reconstructed soils was 3.05, 3.42, 3.64, and 3.40 μmol m^−2^ s^−1^, respectively, and increased during the precipitation event to 5.22, 5.69, 6.36, and 5.79 μmol m^−2^ s^−1^, respectively; it decreased after five days of precipitation to 4.44, 4.82, 5.16, and 4.87 μmol m^−2^ s^−1^, respectively. Precipitation significantly stimulated soil respiration rate of all reconstructed soils. During the observation period, the respiration rate of all reconstructed soils increased before precipitation, and after precipitation it decreased until reaching the soil respiration rate levels before precipitation. The respiration rate of meteorite, shale, sand, and soft rock reconstructed soils increased by 23.2%, 19.3%, 28.1%, and 19.5% relative to one day before precipitation, respectively.

The respiration of meteorite reconstructed soil was significantly lower than the other reconstructed soil in the different periods of precipitation (*p* < 0.05). In contrast, the respiration of shale and soft rock reconstructed soils showed no significant differences (*p* > 0.05).

Figure 2(c) shows the soil respiration for 22 days (i.e. before and after the second precipitation event). During the first five days of the second precipitation event, the average respiration rate of reconstructed soils with added meteorite, shale, sand, and soft rock was 5.86, 6.49, 7.49, and 6.27 μmol m^−2^ s^−1^, respectively, and the average respiration rate during precipitation was 1.70, 1.95, 2.19, and 1.80 μmol m^−2^ s^−1^, respectively; i.e. there was a reduction of approximately 70.9%, 70.0%, 70.8%, and 71.3%, respectively. After five days of precipitation, the average values of soil respiration rate for meteorite, shale, sand, and soft rock reconstructed soils were 4.16, 4.61, 5.32, and 4.45 mol m^−2^ s^−1^, respectively; i.e. relative to the values before precipitation, there was a decrease of 29.3%, 30.2%, 32.6%, and 28.9%, respectively. This event generally inhibited respiration rate of the reconstructed soils, especially during precipitation. During the observation period, respiration rate of all reconstructed soils increased slowly before precipitation, and it showed an upward trend after precipitation, gradually stabilizing. However, the soil respiration rates did not return to the values observed before precipitation. The respiration rate of the meteorite, shale, sand, and soft rock reconstructed soils decreased by 69.8%, 72.7%, 71.0%, and 71.4% relative to one day before precipitation, respectively.

There was no significant difference in respiration among the four reconstructed soils during precipitation (*p* > 0.05). Before and after precipitation, the respiration rates of the sand reconstructed soil were significantly higher than the meteorite reconstructed soil (*p* < 0.05). In contrast, there were no significant differences in respiration rates between the shale and soft rock reconstructed soil (*p* < 0.05).

According to both precipitation analyses, the respiration trends of all reconstructed soils were similar for the two precipitation events, but soil respiration showed different degrees of difference in the different periods (before, during, and after) of precipitation.

### The Relationship between soil respiration and hydrothermal influence factors before, during and after precipitation

#### Soil respiration and soil temperature

The relationship between respiration rate and temperature of all reconstructed soils was consistent with the exponential model (*R*_*S*_ = *a* e^bT^), but the amount of variation in respiration rate explained by temperature was different for the four reconstructed soils (Table 2).

During the second precipitation event, the explanatory power of temperature of all reconstructed soils with respect to the soil respiration rate was less than during the first precipitation event. During precipitation, the temperature of different reconstructed soils had the highest explanatory power, followed by before precipitation, and was lowest after precipitation (the regression coefficient *R*^2^ of the exponential model was expressed as during precipitation > before precipitation > after precipitation). The exponential model was not a good fit for the relationship between temperature and respiration of the four reconstructed soils after the two precipitation events.

#### Soil respiration and soil volumetric water content

The relationship between respiration rate and volumetric water content for all reconstructed soils was consistent with a quadratic polynomial model (*R*_S_ = *a* w^2^ + *b* w + c) (Table 3).

**Table 2:** Relationship between soil respiration rate and soil temperature for reconstituted soil masses at different periods of twice precipitation events.

Reconstituted soil mass types	Rainfall periods	Model parameter (the first rainfall event)				Model parameter (the second rainfall event)			
		a	b	*R*^2^	*p*	a	b	*R*^2^	*p*
Meteorite	Before	0.68	0.10	0.67	<0.001	0.59	0.09	0.52	<0.001
	During	0.32	0.17	0.91	<0.001	0.21	0.11	0.70	<0.001
	After	—	—	—	—	—	—	—	—
Shale	Before	0.57	0.11	0.61	<0.001	0.51	0.10	0.46	<0.001
	During	0.22	0.14	0.78	<0.001	0.10	0.13	0.60	<0.001
	After	—	—	—	—	—	—	—	—
Sand	Before	0.37	0.13	0.61	<0.001	0.47	0.10	0.50	<0.001
	During	0.05	0.16	0.76	<0.001	0.86	0.05	0.63	<0.001
	After	—	—	—	—	—	—	—	—
Soft rock	Before	0.65	0.11	0.74	<0.001	0.60	0.09	0.44	<0.001
	During	0.11	0.12	0.72	<0.001	0.35	0.08	0.69	<0.001
	After	—	—	—	—	—	—	—	—

During the second precipitation event, the soil water content of all reconstructed soils explained the soil respiration rate worse than during the first precipitation event. Compared among the different periods of precipitation, the volumetric water content had the highest ability to respiration of different reconstructed soils, followed by before precipitation, and the lowest after precipitation.

#### Soil respiration, soil temperature and soil volumetric water content

During different periods (before, during, and after precipitation), exponential model of soil respiration rate and soil temperature (Table 2) and a quadratic curve model of soil respiration rate and soil volumetric water content (Table 3) can be used to determine the main factors affecting the respiration of reconstructed soils. It can be seen that the second period of the reconstructed soil was lower than the determinable coefficient of the first precipitation event in the same period (Table 4), indicating that the power-exponential model (two-factor model) had a stronger ability to represent the first precipitation event than the second precipitation event, which was the same as the two single-factor models of temperature (exponential model) and volumetric water content (binomial model) characterizing soil respiration rate changes.

**Table 3:** Relationship between soil respiration rate and soil volumetric water content for reconstituted soil masses at different periods of twice precipitation events.

Reconstituted soil mass types	Rainfall periods	Model parameter (the first rainfall event)					Model parameter (the second rainfall event)				
		a	b	c	*R*^2^	*p*	a	b	c	*R*^2^	*p*
Meteorite	Before	−836.50	264.07	−208.07	0.58	<0.001	−82.28	71.40	20.99	0.55	<0.001
	During	−247.60	154.30	21.75	0.91	<0.001	−631.07	585.87	138.10	0.71	<0.001
	After	−256.40	130.52	−11.44	0.87	<0.001	−6.21	6.51	5.73	0.62	<0.001
Shale	Before	−478.45	141.87	105.20	0.57	<0.001	−54.00	49.64	17.64	0.55	<0.001
	During	−940.88	154.30	21.75	0.92	<0.001	−7.81	1.33	4.70	0.60	<0.001
	After	−124.88	71.38	−4.31	0.82	<0.001	−64.83	52.51	−6.17	0.59	<0.001
Sand	Before	−4380.50	1439.80	−112.17	0.55	<0.001	−72.48	67.90	22.18	0.53	<0.001
	During	−643.70	401.18	−56.55	0.91	<0.001	−35.30	30.11	9.13	0.61	<0.001
	After	−544.00	272.68	−28.14	0.88	<0.001	−55.86	43.39	−3.27	0.51	<0.001
Soft rock	Before	−265.65	885.83	738.72	0.58	<0.001	−24.59	18.23	2.93	0.53	<0.001
	During	−495.20	308.60	−43.50	0.93	<0.001	28.33	−38.52	14.54	0.73	<0.001
	After	−374.12	196.61	−20.12	0.88	<0.001	−89.01	78.9	−12.97	0.70	<0.001

**Table 4:** Relationship between soil respiration rate and soil temperature and soil volumetric water content for reconstituted soils at different periods of twice precipitation events.

Reconstituted soil mass types	Rainfall periods	Model parameter (the first rainfall event)					Model parameter (the second rainfall event)				
		a	b	c	*R*^2^	*p*	a	b	c	*R*^2^	*p*
Meteorite	Before	0.54	0.09	0.26	0.59	<0.001	0.19	0.16	0.36	0.43	<0.001
	During	14.73	0.12	0.28	0.98	<0.001	0.13	0.09	−1.20	0.90	<0.001
	After	11.06	0.10	0.25	0.77	<0.001	5.20	−0.02	−0.17	0.45	<0.001
Shale	Before	0.63	0.11	0.49	0.53	<0.001	0.29	0.13	0.09	0.36	<0.001
	During	0.02	0.28	0.87	0.96	<0.001	0.17	0.09	−0.62	0.68	<0.001
	After	16.80	0.11	0.28	0.62	<0.001	17.96	−0.07	−0.55	0.35	<0.001
Sand	Before	0.64	0.13	0.09	0.54	<0.001	0.50	0.09	−0.14	0.45	<0.001
	During	5.86	0.43	1.25	0.94	<0.001	0.63	0.03	−0.61	0.77	<0.001
	After	6.99	0.26	2.45	0.78	<0.001	1.42	0.07	0.07	0.42	<0.001
Soft rock	Before	0.67	0.10	0.03	0.72	<0.001	0.19	0.15	0.24	0.49	<0.001
	During	12.23	0.10	0.22	0.96	<0.001	0.29	0.03	−1.86	0.85	<0.001
	After	10.64	0.22	1.65	0.78	<0.001	4.22	0.01	0.22	0.20	<0.001

Models of two single factors and both factors explaining reconstructed soils respiration were compared for the different periods of precipitation (before, during, and after). The temperature before the first precipitation event was the main influencing factor for respiration of meteorite, shale, sand, and soft rock reconstructed soils, explaining 67%, 61%, 61%, and 64% of the soil respiration rate variation, respectively. Alternatively, the combination of temperature and moisture during precipitation explained 98%, 96%, 94%, and 96% of the soil respiration rate variation in meteorite, shale, sand, and soft rock reconstructed soils, respectively. After precipitation, water was the main influencing factor of soil respiration, explaining 87%, 82%, 88%, and 88% of the soil respiration rate variation in meteorite, shale, sand, and soft rock reconstructed soils, respectively. Before the second precipitation event, soil volumetric water content was the main factor influencing respiration of the meteorite, shale, sand, and soft rock reconstructed soils, explaining 55%, 55%, 53%, and 53% of the soil respiration rate variation, respectively. During the precipitation event, the respiration of meteorite, shale, sand, and soft rock reconstructed soils was affected by temperature and soil volumetric water content, which explained 90%, 68%, 77%, and 85% of the soil respiration rate variation, respectively. After precipitation, soil volumetric water content was the main factor influencing respiration of meteorite, shale, sand, and soft rock reconstructed soils, explaining 62%, 59%, 51%, and 70% of soil respiration rate variation, respectively.

## Discussion

Our findings are in line with several previous studies which have demonstrated that there were many factors that could influence the response of soil respiration to precipitation events, such as antecedent soil moisture and precipitation conditions^10^, different soil temperature and moisture conditions^11^, temperature sensitivity^12^, and different precipitation^13^. In addition, the different natural characteristics of the added materials was an important factor that could influence the response of soil respiration to precipitation.

The moisture and precipitation of reconstructed soils were lower before the first precipitation event, which caused soil respiration to show an upward trend during precipitation, while soil moisture and precipitation were higher before the second precipitation, and the soil respiration showed a decreasing trend during precipitation. Our findings are consistent with several previous studies, which have indicated that a precipitation pulse significantly increased soil respiration after continuous drought^10,29^. Increased precipitation may not always increase soil respiration, and soil respiration responded nonlinearly to increased precipitation^11^. The nonlinear increases resulted from the wet initial water conditions that reduced or even completely masked the pulse effect^30^. Soil moisture conditions before precipitation play an important role in regulating the responses of soil respiration to precipitation. Because the antecedent water conditions and precipitation determine the soil moisture environment. When the soil environment is dry, the sensitivity of soil moisture is high, and the soil respiration response is strong when precipitation occurs. Under humid conditions, the soil water sensitivity is low, and the soil respiration response is not strong.

Soil temperature and moisture during all three precipitation periods of the first precipitation were both lower than those during the second precipitation event. Different soil temperature and moisture conditions can result in different rates of microbial activity, and microbial activity can also affect soil respiration and its response to precipitation events^12,31,32^. Temperature and moisture are the main driving factors of the change in the soil microbial community, and there are thresholds for the effects of temperature and moisture on soil microorganisms. The microbial activity intensifies with increasing temperature in a certain temperature range. Too much or too little water can cause soil microbial activity to decrease. In addition, the underlying mechanisms that can also affect the soil microbial community include, adding different materials, different physical indicators such as soil permeability and structure, and different environments in which microorganisms live. Under dry conditions, the microorganisms are dormant due to low water availability. The occurrence of precipitation will activate the microorganisms, while water will occupy the soil pores, Microbes respond to the strong osmotic shock and their carbon-containing compounds are discharged out of the cells and decomposed by microorganisms generating a large amount of carbon dioxide^33^, and the soil respiration will quickly strengthen. In humid conditions, precipitation can cause excessive soil moisture, where the soil is in an anaerobic state, and plant roots and microbial activity are limited, and soil respiration is inhibited.

The temperature sensitivity of soil respiration during the first precipitation event was greater than during the second precipitation event. However, the first precipitation was lower than the second. This phenomenon was consistent with several previous studies which indicated that increased precipitation can also have a nonlinear impact on the temperature sensitivity of soil respiration^10^. Different precipitation events can result in different responses of plant production, and plant production affects soil respiration^31,34^. This is because increased precipitation significantly increases environmental humidity, which is beneficial to plant growth^34^. The different natural characteristics of the added materials can result in different responses of soil temperature and moisture to precipitation, and soil temperature and moisture can affect soil respiration. Previous studies have shown that soft rock and meteorite were materials that retain water, whereas loose sandy structures did not retain water and did not heat up quickly^14–20^. This was confirmed by the order of the temperature and water content of the four reconstructed soils for the different precipitation events in this study.

Under the influence of the above factors, the response of soil respiration on the first and second precipitation event was different. The results of this study showed that the first precipitation event significantly increased the soil respiration rate of the different reconstructed soils; this is in accordance with previous studies^35^. The first precipitation event triggered soil respiration, which lasted for five days, similar to the length of the respiration pulse reported in a previous study (2 to 6 days)^36^. This can be related to one or more of the following mechanisms. First, during the first precipitation, the soil volumetric water content increased, occupying the soil pores, increasing the amount of carbon dioxide emissions, and inducing soil respiration; Second, after the first precipitation event, the temperature increased, the soil moisture decreased, and the organic matter was more closely bonded to the soil aggregate, the decomposition of microorganisms was weakened and the contribution to soil respiration was reduced^37^.

However, some researchers believe that in relatively humid soils, the soil respiration rate is inhibited^10,38,39^, which is consistent with the conclusions derived from the observations based on the second precipitation event in this study. One or more of the following mechanisms could explain this phenomenon. First, the second precipitation event was characterized by heavy precipitation and lasted a long time. Five days after precipitation, the soil respiration had not recovered to the level before precipitation. The soil moisture decreased slowly after precipitation. The water occupied most of the soil pores and soil respiration was inhibited; Second, the second precipitation event decreased the soil temperature, which fluctuated greatly after precipitation, increasing the soil respiration recovery time. Therefore, the soil respiration rate recovery time was longer^40^.

The variation in reconstructed soils respiration before, after, and during the first precipitation event were explained by soil temperature, soil moisture, and both factors together, respectively. This was because the magnitude of change in soil temperature and moisture can affect the soils respiration response under various environmental conditions^10,11,41,42^. In a previous study, researchers set three warming treatments and found that the variation in soil respiration was explained by soil temperature for the control plots but was explained by soil moisture for the high warming treatment. Meanwhile, soil temperature and moisture together explained the variation in respiration for the low warming treatment^41^. These results also explained the above conclusion of this study.

In this study, the soil moisture changed significantly before the first precipitation event, water was the main influencing factor, soil water content was relatively stable before the second precipitation event, and soil temperature was the main influencing factor. This was because three small-scale precipitation events occurred before the second precipitation event, with 18.8, 4.4, and 15.5 mm rain falling. The soil volumetric water content was in the recovery period from the last precipitation and decreased continuously. Therefore, soil moisture became the main factor affecting the respiration rate before the second precipitation event. Importantly, the soil was dry before the first precipitation event. These results are consistent with the results of previous^43,44^. In addition, the second precipitation amounts were relatively larger, and the inhibitory effect on soil respiration was longer. Our findings are consistent with several previous studies which indicated that the amount of precipitation determines how much soil temperature and moisture influence soil respiration^45,46^.

## Conclusions

1. Both precipitation events significantly reduced soil temperature. During the observation period, the temperature of four reconstructed soils increased before and after both precipitation events, and soil temperature decreased during precipitation. The temperature fluctuated greatly after the second precipitation event.
2. Precipitation improved the water content of the reconstructed soils. During the observation period, the volumetric water content of all reconstructed soils increased during both precipitation periods, and gradually decreased after precipitation. The soil volumetric water content before the first precipitation event was relatively stable, and gradually decreased before the second precipitation event.
3. The first precipitation event significantly stimulated respiration rate of different reconstructed soils. During the observation period, the respiration rate of four reconstructed soils increased before precipitation and showed a decreasing trend afterwards. Moreover, the rate of decline increased until it reached the level of soil respiration rate before precipitation, and soil respiration rate increased during precipitation. The second precipitation event generally inhibited respiration rate in all reconstructed soils, especially during the precipitation event. Before precipitation, the respiration rate of all reconstructed soils increased slowly, showing an upward trend, and gradually stabilizing after precipitation. However, it did not return to the soil respiration rate before precipitation.
4. According to the exponential model of respiration rate and temperature for four reconstructed soils, the quadratic curve model of soil respiration rate and volumetric water content, and the power-exponential model soil respiration rate with soil temperature and moisture, these parameters can be considered as the main factors affecting soil respiration rates in different periods of both precipitation events. The change of soil volumetric water content before precipitation can be considered the main influencing factor of soil respiration rate change. The soil volumetric water content changed significantly before precipitation, water is the main influencing factor, soil water content is relatively stable, soil temperature is the main influencing factor, and the precipitation process is affected by soil temperature and moisture. After precipitation, water is the main influencing factor of soil respiration rate.
